# Bilateral discoid medial meniscus associated with meniscal tears and hypoplasia of the medial femoral condyle: A case report

**DOI:** 10.1097/MD.0000000000008637

**Published:** 2017-11-17

**Authors:** Hong-De Wang, Shi-Jun Gao

**Affiliations:** a Department of Orthopedics, Third Hospital of Hebei Medical University; b Orthopaedic Biomechanics Laboratory of Hebei Province, Shijiazhuang, Hebei, People's Republic of China.

**Keywords:** arthroscopy, case report, discoid medial meniscus

## Abstract

**Rationale:**

Bilateral discoid medial menisci is an extremely rare abnormality of the knee joint. The presence of a discoid medial meniscus has been associated with magnetic resonance imaging (MRI) and radiographic changes in the tibial region, such as cupping of the medial tibial plateau and tibial physis collapse. While discoid medial meniscal tears with hypoplasia of the femoral medial condyles have not been previously reported. Herein, we report a case of bilateral discoid medial menisci associated with meniscal tears and femoral bone changes.

**Patient concerns:**

A 28-year-old man presented with left knee pain and restricted range of motion; the right knee was asymptomatic.

**Diagnoses:**

Based on radiographic and MRI findings, he was diagnosed with bilateral discoid medial meniscal tears.

**Interventions:**

Partial meniscectomy and reshaping were performed for the torn discoid medial meniscus of the left knee only.

**Outcomes:**

MRI revealed short, flattened femoral medial condyles in the coronal and sagittal planes, and hypoplasia of the femoral medial condyles in the axial plane; these findings were confirmed arthroscopically in the left knee. The patient had a satisfactory results at the 12-month follow-up.

**Lessons:**

This case indicates a potential link between discoid medial menisci and hypoplasia of the femoral medial condyle. We recommend preservation of the discoid medial meniscus in asymptomatic patients, while arthroscopic partial meniscectomy and reshaping is recommended in symptomatic patients.

## 1 Introduction

Discoid medial meniscus is an extremely rare abnormality of the knee, with an estimated incidence of 0.12%.^[1]^ Cases of bilateral discoid medial menisci are even rarer.^[2]^ The first case of a discoid medial meniscus was reported in 1941,^[3]^ and fewer than 30 cases of bilateral discoid medial menisci have been reported since then.^[4–9]^ The presence of a discoid medial meniscus has been associated with magnetic resonance imaging (MRI) and radiographic changes in the tibial region, such as cupping of the medial tibial plateau and tibial physis collapse.^[7,10]^ In contrast, discoid medial meniscal tears with bone changes in the femoral region have not been previously reported.

Herein, we present a case of bilateral discoid medial menisci with medial meniscal tears and associated hypoplasia of the femoral medial condyles detected by MRI and successfully treated by arthroscopy. Informed consent was obtained from the patient for the publication of this case report. This case report was approved by ethics committee of our hospital (Institutional Review Board of The Third Hospital of Hebei Medical University).

## 2 Case report

A 28-year-old man experienced medial left knee pain, effusion, and locking of his left knee for 12 days. He had a history of left knee sprain while walking fast 12 days ago. There was no other history of left knee injury.

On physical examination, the range of motion of the left knee was restricted due to pain from 0° to 100°. There was mild left knee joint effusion, medial joint line tenderness, and the McMurray test was positive. No ligament instability was noted. The right knee was asymptomatic.

**Figure 1:**
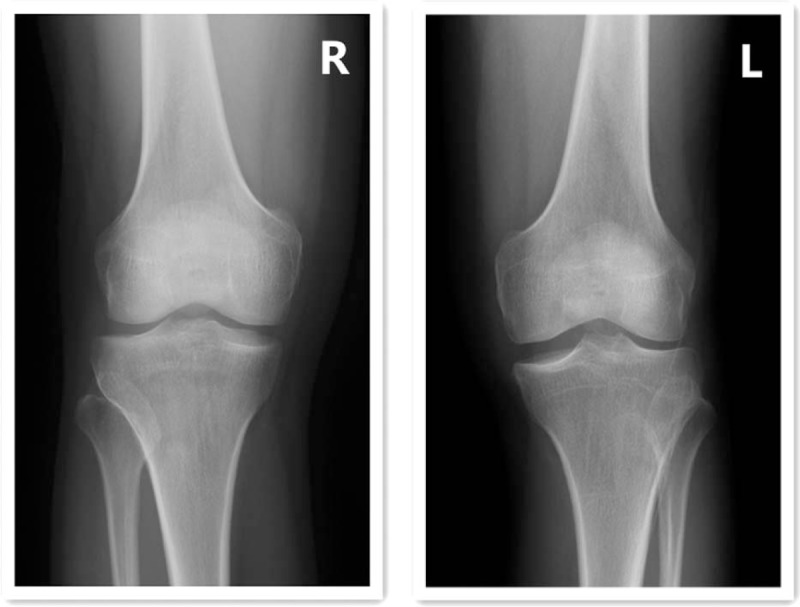
Standing anteroposterior radiographs of both knees. Radiography showed widened medial joint spaces, squaring of the medial femoral condyles, and cupping of the medial tibial plateaus bilaterally.

**Figure 2:**
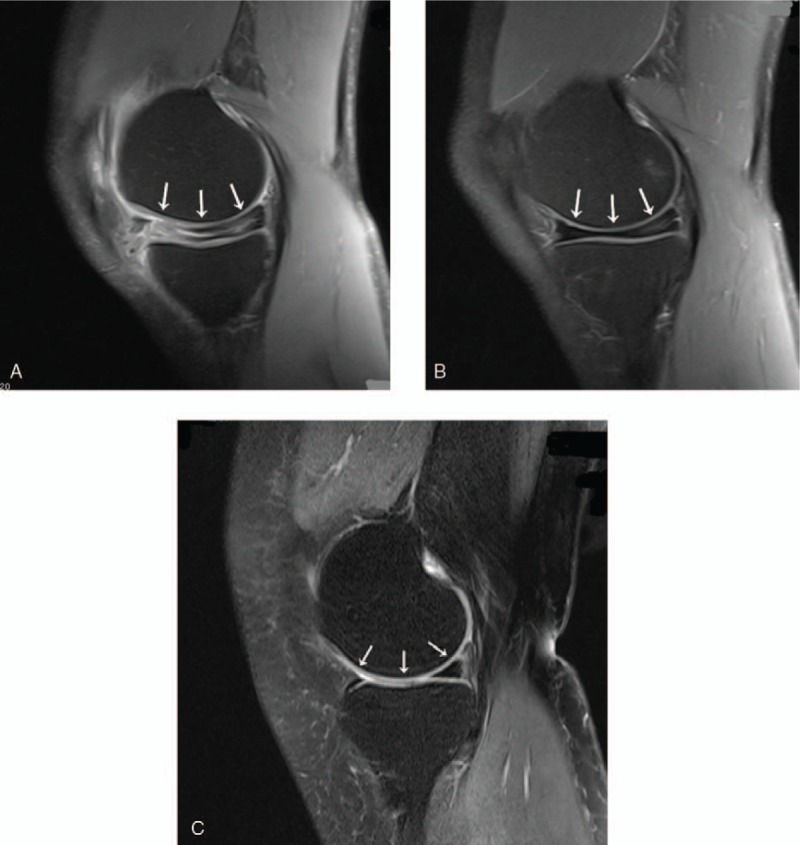
MRI images of the patient (A, B) and a normal knee (C) in the sagittal plane. (A) T2-weighted sagittal MRI of the left knee showing the discoid medial meniscus with a horizontal and bucket-handle-like tear. (B) Discoid medial meniscus with grade II injury in the right knee. (C) Normal medial meniscus. (A, B) The distal and posterior sections of the femoral medial condyles were more flattened (indicated by the white arrows) than normal (C). MRI = magnetic resonance imaging.

**Figure 3:**
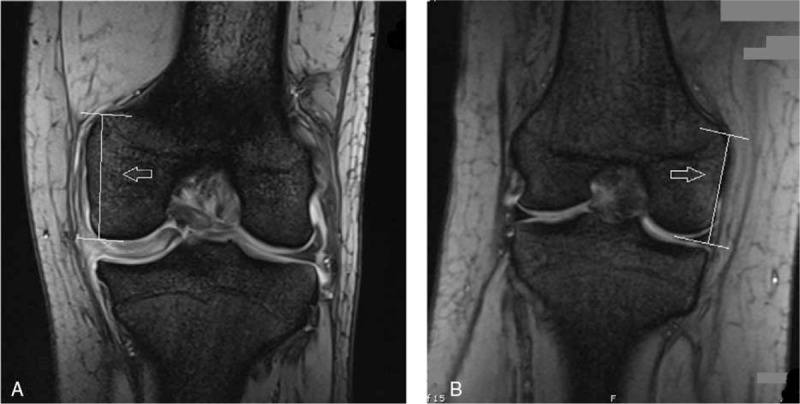
MRI image of the patient (A, left knee) and a normal knee (B, right knee) in the coronal plane. Relative to the height of the lateral femoral condyle, the patient's femoral medial condyle is shorter than would be expected in a normal knee (indicated by the white arrow). MRI = magnetic resonance imaging.

**Figure 4:**
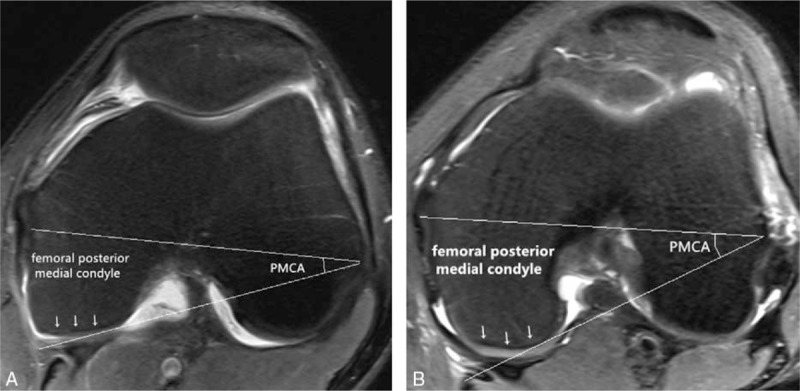
MRI images of the patient (A) and a normal knee (B) in the axial plane. The posterior medial condyle angle (PMCA) of the current patient was smaller than normal. The articular surface of the posterior medial condyle is obviously flatter than in the normal knee. These findings suggest hypoplasia of the femoral posterior medial condyle. MRI = magnetic resonance imaging.

Standing anteroposterior radiography showed a widened medial joint space, squaring of the medial femoral condyle, and cupping of the medial tibial plateau in both knees (Fig. 1). So both knees were examined by MRI, which revealed bilateral discoid medial menisci. The left medial meniscus had a horizontal and bucket-handle-like tear (Fig. 2A), the right side had a grade II injury (Fig. 2B). The bilateral lateral menisci were normal. MRI suggested hypoplasia of the femoral medial condyles bilaterally. The height of both femoral medial condyles was shorter than normal in the coronal plane (Fig. 3). The distal and posterior portions of both femoral medial condyles were flattened, especially in the distal femoral medial condyles in the sagittal plane (Figs. 2A, B and 3A). On axial view, the bilateral posterior medial condylar angles^[11]^ were smaller than normal (Fig. 4).

**Figure 5:**
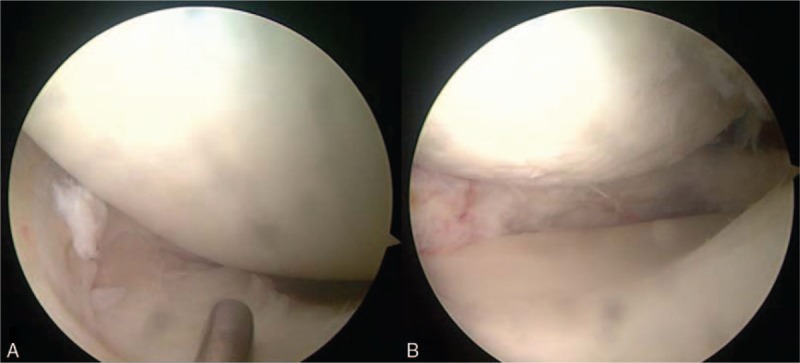
Arthroscopic images of the left knee. (A) Arthroscopic examination showed a complete tear with a horizontal and bucket-handle-like tear. (B) The medial meniscus after partial meniscectomy and reshaping. Cartilage degeneration was seen on the femoral medial condyle.

Arthroscopic examination of the left knee confirmed the presence of a discoid medial meniscus (Fig. 5A). The medial meniscus was complete and had a horizontal and bucket-handle-like tear extending from the anterior horn to the meniscal body, and displacing to the intercondylar area; the lateral meniscus was normal. Cartilage degeneration was seen on the femoral medial condyle (Fig. 5B). There was also a pathologic medial patellar plica with thickening, leading to meniscal compression. We performed partial meniscectomy of the complete discoid medial meniscus of the left knee and resection of the pathologic medial patellar plica (Fig. 5B). As the right knee was asymptomatic, it was not operated on.

Full weight-bearing and full range of motion were allowed from postoperative day 1, as much as could be tolerated. Full contact sports were allowed 3 months postoperatively. The patient was satisfied with the outcome and was asymptomatic with a completely painless full range of motion at the 12-month follow-up. Physical examination revealed no joint line tenderness, negative McMurray testing, and no limitation of motion.

## 3 Discussion

The incidence rate for discoid lateral meniscus is reportedly 1.5%.^[12]^ Discoid medial meniscus is rarer, with an estimated incidence of 0.12%,^[1]^ and bilateral discoid medial menisci are rarer still.^[2]^ The real incidence of discoid medial menisci is difficult to ascertain, as an unknown percentage of discoid menisci may be asymptomatic. Symptomatic tear of the discoid lateral meniscus is a well-known injury that mainly affects the young. However, it is uncommon for a discoid meniscus to become symptomatic and tear in adults. Symptomatic discoid lateral meniscus tear occurs mainly in young and middle-aged women in the Chinese population.^[13]^

The etiology of discoid menisci has not yet been completely ascertained. Smillie^[14]^ postulated that the discoid shape is normal in the developing embryo, and that the discoid shape is sometimes retained due to failure of absorption of the central part during the fetal stage. Kaplan^[15]^ stated that the meniscofemoral ligament of Humphrey plays an important role in discoid meniscus formation, as the meniscus becomes hypermobile due to changes in the attachment of the posterior coronary ligament.

MRI is currently the recommended non-invasive method for diagnosis of discoid meniscus. A discoid meniscus is suggested by the presence of the “bow tie” sign in >3 slices in the sagittal plane. However, the most accurate criterion for diagnosis is a ratio of the minimal meniscus width to the maximal tibial width of >20% in the coronal plane.^[15]^ Wantanabe et al^[16]^ classified the lateral meniscus into 3 types: complete, incomplete, or Wrisberg variant (lacking a posterior coronary ligament and capsular attachments); however, there is still no classification for discoid medial menisci. The current patient had a complete discoid medial meniscus of the left knee.

Many authors have reported bone changes associated with the presence of a discoid medial meniscus, especially in the tibial region. Weiner and Rosenberg^[10]^ described a case of discoid medial meniscus associated with radiographic irregularity and proximal mediotibial physis collapse. Atay et al^[7]^ reported increased concavity of the medial tibial plateau in a bilateral discoid medial meniscus case, and MRI showed some depression of the medial tibial plateau and decreased signal intensity of the subchondral medial tibial epiphysis consistent with reactive sclerosis. In contrast, few authors have reported changes in the femoral bone associated with a discoid meniscus. In the current case, the femoral medial condyle was shorter and flatter than normal in the coronal plane. Xu et al^[11]^ reported that hypoplasia of the posterior lateral femoral condyle is typically seen in patients with complete discoid lateral meniscus, as determined by measuring the posterior lateral condylar angle and the posterior medial condylar angle on MRI. Similarly, the current case had hypoplasia of the posterior medial femoral condyle on axial MRI. Kamei et al^[17]^ reported that a discoid lateral meniscus may result in excessive stress on the lateral femoral condyle and affect ossification of the secondary epiphyseal nucleus, and Deie et al^[18]^ reported that the mechanism leading to osteochondritis dissecans of the lateral femoral condyle might be related to the type and stress of the lateral meniscus. Therefore, the mechanical stress in the lateral femoral condyle varies in cases of complete and incomplete discoid lateral menisci. It remains unproven whether these changes occur in other discoid medial meniscus cases. We speculate that the reason for such bone changes may be due to the discoid shape, which decreases stress on the femoral medial condyle during the process of growth.

In line with the preference of most surgeons,^[5,6,10]^ we chose to perform partial meniscectomy and reshaping instead of complete removal of the meniscus, as reshaping can provide a stable peripheral attachment.^[4,19]^ Furthermore, the axial alignment of the lower limb with a torn discoid meniscus is altered after arthroscopic meniscectomy. Wang et al^[20]^ reported that the vagus inclination was more pronounced in patients with a torn discoid lateral meniscus compared with those with a non-discoid lateral meniscus. So, we speculate that total meniscectomy for torn discoid medial meniscus may increase the varus inclination of the lower limb and the stress on the tibiofemoral articular surface due to hypoplasia of the medial femoral condyle. The current patient was asymptomatic at the 12-month follow-up. Although MRI showed injury of the discoid medial meniscus of the right knee, there were no associated symptoms. Therefore, we recommend that the discoid medial meniscus should be preserved if symptoms are absent, and we believe that partial meniscectomy can achieve a stable rim and good clinical outcome.

In conclusion, discoid medial meniscus is a rare abnormality that is best diagnosed via non-invasive MRI. Bone changes, especially hypoplasia of the femoral medial condyle, may be related to discoid medial menisci. We recommend preservation of the discoid medial meniscus in asymptomatic patients, and arthroscopic partial meniscectomy and reshaping in symptomatic patients.

## Acknowledgment

The authors thank Fu-Shun Wang and Xiao-Jing Wang for their assistance in editing figures.
